# Fetal cephalohematoma following external cephalic version: a case report

**DOI:** 10.1186/s12884-026-09668-z

**Published:** 2026-07-20

**Authors:** Jiangyan Xie

**Affiliations:** Department of Obstetrics, Hainan Women and Children Medical Center, Haikou, China

**Keywords:** Breech presentation, External cephalic version, Fetal cephalohematoma, Case report

## Abstract

**Background:**

External cephalic version (ECV) is an obstetric procedure that involves manually turning the fetus inside the pregnant woman’s abdomen to change a breech or transverse presentation to a cephalic presentation. It can significantly increase the proportion of cephalic presentations during delivery and reduce the cesarean section rate for term singleton breech presentations, making it a relatively safe operation. In this case, we report a case of fetal cephalohematoma following ECV, which has not been previously documented in the literature.

**Case presentation:**

A 33-year-old multiparous woman with a breech presentation underwent an ECV at 39 weeks and 1 day of gestation. Nifedipine was taken orally before the procedure to inhibit uterine contractions, and combined spinal-epidural anesthesia was performed. ECV was successful on the third attempt. On the 10th postoperative day, an ultrasound examination revealed a fetal cephalohematoma, and a cesarean section was eventually performed. The fetal cephalohematoma gradually resolved after birth, without causing severe adverse effects on the newborn.

**Conclusion:**

ECV has few complications and can significantly reduce the cesarean section rate in breech presentations. However, during the procedure, it is important to strengthen perioperative management and improve the operator’s professional skills to reduce the risk of complications. The procedure should be performed gently, without unnecessary force.

## Background

Breech presentation is a common fetal malpresentation during pregnancy, occurring in approximately 3–4% of term pregnancies [1]. A multicenter randomized controlled study published in The Lancet in 2000 changed the choice of delivery methods for singleton term breech pregnancies worldwide, with an increasing number of breech presentations opting for cesarean rather than vaginal breech delivery [1]. The rise of cesarean section rates and the associated complications [2] and increased economic costs [3] have led to a re-evaluation of delivery methods for breech presentations. External Cephalic Version (ECV) is an obstetric operation that involves manually turning the fetus inside the pregnant woman’s abdomen to change a breech or transverse presentation to a cephalic presentation [4]. Due to its few complications and significant reduction in the cesarean section rate for term breech pregnancies, it is widely recommended by the American College of Obstetricians and Gynecologists (ACOG) [5] and the Royal College of Obstetricians and Gynecologists (RCOG) [3].

ECV can significantly increase the proportion of cephalic presentations during delivery, reduce the cesarean section rate for term singleton breech presentations, and does not increase adverse neonatal outcomes, making it a relatively safe procedure. Mild complications occur in less than 5% of ECVs, including transient changes in fetal heart rate, mild antepartum bleeding, and premature rupture of membranes, while more severe complications such as placental abruption or fetal distress requiring emergency cesarean section occur in less than 1% of cases [6]. In this case, the patient developed a fetal cephalohematoma after ECV. To the best of our knowledge, this complication has not been reported previously.

## Case presentation

A 33-year-old G4P2 woman with two previous spontaneous vaginal deliveries of newborns weighing 3000 g and 3850 g, respectively. At 33 weeks of pregnancy, an ultrasound examination revealed a breech presentation. The patient declined intervention at that time and opted for expectant management. At 39 weeks and 1 day, she was admitted to the hospital for a planned cesarean section. On examination: breech presentation, estimated fetal weight 3400 g, non-stress test normal. Ultrasound showed: left sacroanterior (LSA) position, frank breech presentation, placenta located on the anterior wall, one loop of nuchal cord, maximum amniotic fluid depth 43 mm, amniotic fluid index (AFI) 116 mm. After being informed of the delivery options, including vaginal breech delivery, vaginal delivery after ECV, and cesarean section, and after full counseling on the associated risks and alternatives, the patient opted to undergo ECV.

The ECV was performed by an experienced attending obstetrician, who has performed over 50 ECV procedures. The department performs approximately 30–40 ECVs per year with a success rate of about 70–75%. The procedure was performed under combined spinal-epidural anesthesia. The patient received 10 mg of nifedipine orally before the procedure to inhibit uterine contractions (administered according to our hospital’s routine protocol). Ultrasound confirmed LSA position with fetal heart rate 146 bpm. The operator stood on the patient’s right side. Both hands lifted the fetal buttocks out of the pelvis, with the right hand holding the buttocks and the left hand holding the head. Two counterclockwise attempts were unsuccessful. On the third attempt, a clockwise rotation successfully turned the fetal head to the pubic symphysis. The operator subjectively noted considerable resistance during the procedure and therefore applied greater force. The total procedure time was approximately 8 min. Ultrasound confirmed right occiput posterior position, fetal heart rate 160–170 bpm, no placental abruption, and two loops of nuchal cord (increased from one loop pre-procedure). This finding was discussed with the patient, and she opted for expectant management with close monitoring.

Postoperatively, folded towels were placed on both sides of the abdomen and secured with a belly band. Continuous fetal heart rate monitoring showed normalization to 140 bpm after 5 min. The patient had no vaginal bleeding or fluid leakage. On the first postoperative day, follow-up ultrasound showed right occiput posterior position, one loop of nuchal cord, and normal umbilical artery Doppler indices. The patient was discharged.

The patient was readmitted at 41 weeks of pregnancy for a planned late-term induction of labor. After admission, color Doppler ultrasound showed a cystic anechoic mass behind and to the right side of the fetal skull, measuring approximately 42 mm×39 mm, containing fine speckled echoes rolling inside, with no obvious blood flow signals within or around it, suspected to be a cephalohematoma (Fig. 1). The fetal position was right occiput posterior, maximum amniotic fluid depth 24 mm, AFI 71 mm, with one nuchal cord loop. Given the possibility of hematoma enlargement during vaginal delivery, intracranial bleeding, or rupture, the patient elected to undergo cesarean section. The surgeon was gentle, avoiding pressure on the fetal head mass. A live male infant weighing 3700 g was delivered with Apgar scores of 10. A soft, ill-defined mass approximately 60 mm x 60 mm was palpable on the neonatal head (Fig. 2). Umbilical artery blood gas analysis performed immediately after delivery showed a normal pH (7.41). The newborn was transferred to the neonatal department. Coagulation testing after transfer showed no clinically significant abnormalities. Ultrasound confirmed a suspected cephalohematoma (66 mm x 33 mm). The newborn received monitoring for jaundice, vitamin K1 prophylaxis, supportive care (fluids), and antibiotic therapy. The cephalohematoma gradually decreased to approximately 20 mm x 10 mm on the third day after birth and completely resolved by the tenth day. Jaundice did not progress. The mother recovered well and was discharged on the fourth postoperative day.

**Fig. 1:**
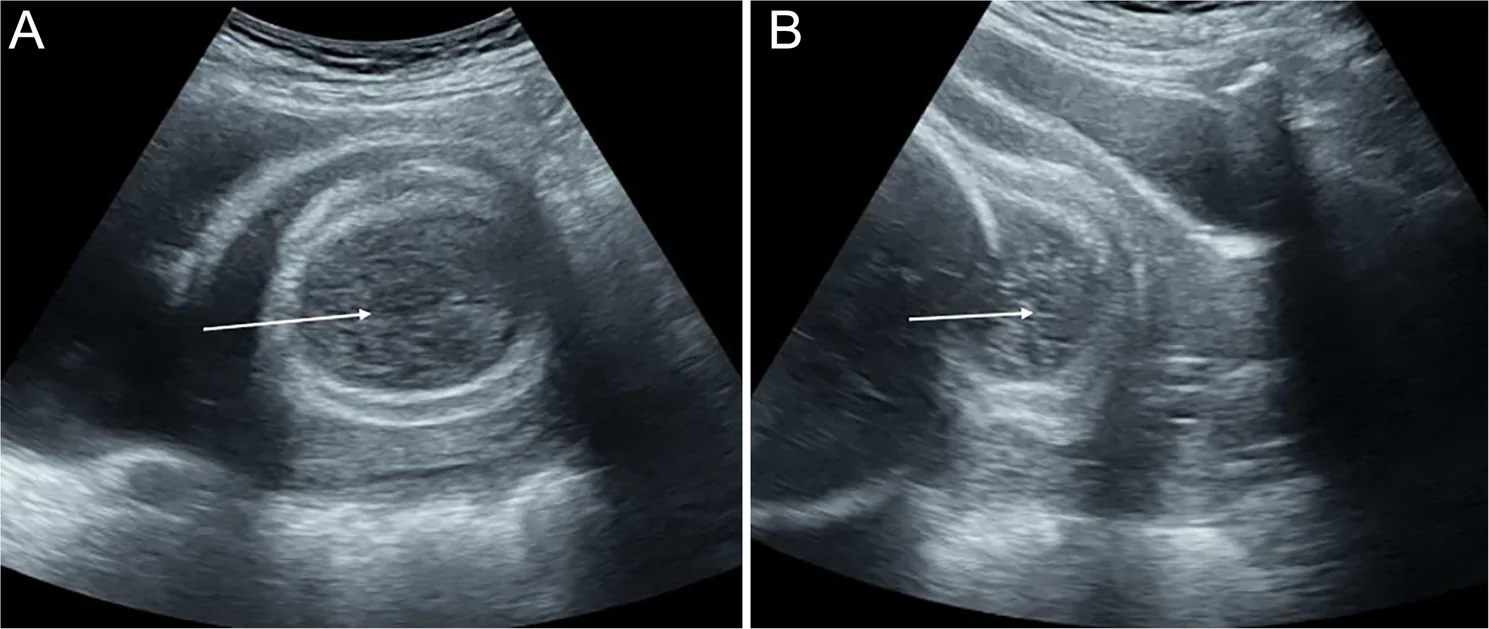
Ultrasound image, arrows point to the fetal cephalohematoma. **A** Cross section; **B** Sagittal plane

**Fig. 2:**
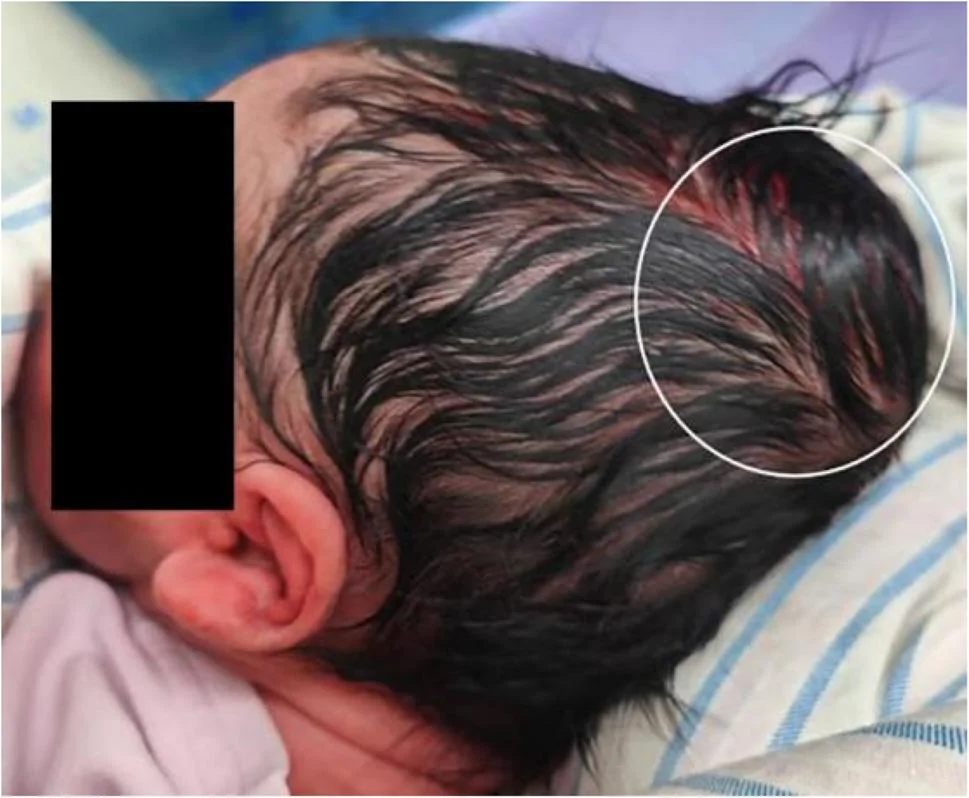
Neonatal cephalohematoma (the infant’s face has been anonymized with a black box to protect privacy)

## Discussion

Complications of ECV include cord presentation, cord prolapse, premature rupture of membranes, abnormal fetal heart rate, fetal distress, fetal fractures, fetomaternal hemorrhage, reversion of fetal position, neonatal asphyxia, intrauterine fetal death, and placental abruption [6]. This case describes the first instance of a fetal cephalohematoma occurring after ECV, although spontaneous antenatal cephalohematomas have been reported [7–9].

Fetal cephalohematoma is caused by compression during delivery, leading to injury of the periosteum and rupture of subperiosteal blood vessels, with blood accumulating between the skull and the periosteum. Its incidence is 1%-4% [10]. Fetal cephalohematoma is often associated with vacuum extraction and forceps-assisted delivery [11], with occasional reports of spontaneous occurrence [12]. Ultrasound is the preferred method for assessing fetal cephalohematoma, including location, size, shape, texture, and vascularity [13]. In this case, the fetal cephalohematoma appeared after ECV, was discovered by ultrasound, and postpartum assessment also relied on ultrasound. No cephalohematoma was seen on the first post-ECV day, but it was found on day 10. Because no other identifiable cause was present, we speculate that the application of considerable pressure to the fetal head during ECV may have contributed to the hematoma; however, the 10-day interval between ECV and diagnosis is a limitation, and spontaneous occurrence cannot be entirely excluded. Most cephalohematomas appear 2–3 days after delivery and generally require no special treatment, with over 80% resolving within 3–4 weeks. Some may take longer, and hematoma absorption can worsen jaundice. If not absorbed over a long period, ossification may occur, requiring surgical treatment [14, 15]. Rarely, hematomas can become infected [16], necrose, or cause skull destruction [9]. In this case, the newborn’s cephalohematoma significantly decreased by day three and completely resolved by day ten. Although it did not cause adverse effects, obstetricians should be aware that a fetal cephalohematoma may occur after ECV, and consider post‑procedural ultrasound evaluation of the fetal head. Coagulation profile was essentially normal, excluding coagulopathy as a cause of the cephalohematoma.

The RCOG believes that taking appropriate preventive measures may help reduce ECV complications [5]. ECV should be performed in institutions capable of emergency cesarean section, A trained practitioner is an important predictor of ECV success [17]. In the present case, we adhered to these principles: ECV should be performed in a tertiary center with emergency cesarean section capability; the operator had completed over 20 ECV procedures under supervision; pre-procedural ultrasound and electronic fetal heart rate monitoring were performed; written informed consent was obtained from the patient; and if vaginal bleeding, unexplained or disproportionate pain (in procedures without neuraxial anesthesia), or persistent fetal heart rate abnormalities occur, emergency cesarean section is recommended. In the present case, no vaginal bleeding, abnormal pain, or persistent fetal heart rate abnormalities occurred; therefore, emergency cesarean section was not required.

Regarding the number of attempts allowed for ECV, the RCOG recommends no more than four attempts, with the entire procedure not exceeding 10 min [6]; the ACOG practice guidelines do not explicitly specify a maximum number of attempts [3]. A large retrospective study reported spontaneous re‑version to a breech position in approximately 2% of successful ECVs [18]. The ACOG recommends considering repeat ECV if the fetus returns to breech [13]. In the present case, external cephalic version was successfully achieved on the third attempt, which is within the recommended limit, and no reversion occurred.

Regarding timing, ACOG recommends performing ECV at 37 weeks [3]; RCOG similarly suggests offering it at term (37 weeks) [5]. In this case, ECV was performed at 39 weeks and 1 day, and no prematurity-related risks were involved.

Literature reports that predictive factors associated with successful ECV include multiparity, non-engagement of the presenting part, use of tocolytic agents, palpable fetal head, low maternal body weight, posterior placenta, frank breech presentation, and an amniotic fluid index > 10 cm [19, 20]. In this case, the patient was multiparous, had a palpable fetal head, an AFI of 11.6 cm, and received tocolytic agents during the procedure–all factors favoring ECV success. However, the patient also had some factors that may reduce the success rate, such as a BMI of 30.2 (obese) and an anterior placenta (rather than posterior). After comprehensive consideration, the patient and her family still opted to attempt ECV.

Contraindications to ECV include multiple pregnancies, ruptured membranes, severe comorbidities, abnormal fetal heart rate, fetal death, recent vaginal bleeding, or absolute indications for cesarean section [21]. Oligohydramnios (deepest vertical pocket [DVP] < 2 cm or AFI < 5 cm) and nuchal cord are relative contraindications. In this case, the patient had a DVP of 4.3 cm and an AFI of 11.6 cm, both within the normal range, so oligohydramnios was not present. Although there was one loop of nuchal cord, the patient opted to undergo ECV after full counseling on the risks.

International standards for anesthesia in ECV are lacking, and various methods including epidural, spinal, inhalation, and intravenous anesthesia have been used to facilitate ECV [22, 23]. Meta-analyses have shown that neuraxial anesthesia combined with tocolytics significantly increases ECV success and vaginal delivery rates [22]. Combined spinal-epidural anesthesia was chosen in this case for its rapid onset and more complete uterine relaxation. Regarding tocolysis, RCOG recommends beta-agonists (e.g., ritodrine, terbutaline) over calcium channel blockers due to stronger evidence [5]. Because nifedipine is orally convenient and has a good safety profile, it is routinely used in our hospital. In this case, nifedipine was administered orally before the procedure along with combined spinal-epidural anesthesia, resulting in no uterine contractions and no pain during the procedure, which contributed to the success of the ECV. However, the evidence for nifedipine is less robust than that for beta-agonists, and this is a limitation.

Notably, this is the first report of a fetal cephalohematoma following ECV, adding a previously undocumented complication to the known safety profile of the procedure. The case was managed in a tertiary center with standardized perioperative protocols, and the diagnosis and resolution of the hematoma were confirmed by serial ultrasound examinations. Despite these strengths, several limitations of this report should be acknowledged. First, as a single case report, the findings are not generalizable and do not establish a causal relationship between ECV and cephalohematoma. Second, the 10‑day interval between the ECV procedure and the ultrasound diagnosis of the cephalohematoma precludes definitive determination of its exact onset, and spontaneous antenatal occurrence cannot be entirely ruled out. Third, the force applied during the ECV was not quantitatively measured, limiting our ability to correlate the degree of pressure with the development of the hematoma. Fourth, this case involved an experienced operator in a tertiary center, which may not reflect the outcomes of less experienced providers or other clinical settings.

## Conclusion

External cephalic version (ECV) is an effective method for reducing the cesarean section rate in breech presentations, but its indications and contraindications require individualized assessment. Adequate pre-procedural preparation, appropriate patient selection, and standardized technique can help reduce complications, including fetal cephalohematoma. The procedure should be performed gently, without unnecessary force. As this case is the first report of fetal cephalohematoma following ECV, we recommend that post-ECV ultrasound, in addition to routine parameters, should also pay attention to individual fetal structures to facilitate early detection of such complications.

## Data Availability

No datasets were generated or analysed during the current study.

## Abbreviations

ECV: External cephalic version
ACOG: American College of Obstetricians and Gynecologists
RCOG: Royal College of Obstetricians and Gynaecologists
LSA: Left sacroanterior
DVP: Deepest vertical pocket
AFI: Amniotic fluid index
